# S105. VALIDATING THE PREDICTIVE ACCURACY OF THE NAPLS-2 PSYCHOSIS RISK CALCULATOR IN A CLINICAL HIGH-RISK SAMPLE FROM THE SHARP (SHANGHAI AT RISK FOR PSYCHOSIS) PROGRAM

**DOI:** 10.1093/schbul/sby018.892

**Published:** 2018-04-01

**Authors:** TianHong Zhang, HuiJun Li, LiHua Xu, YingYing Tang, HuiRu Cui, Junjie Wang, Chunbo Li, Kristen Woodberry, Daniel I Shapiro, Margaret Niznikiewicz, Martha E Shenton, Matcheri S Keshavan, William S Stone, JiJun Wang, Robert W McCarley, Larry J Seidman

**Affiliations:** 1 Shanghai Mental Health Center, Shanghai Jiao Tong University School of Medicine; 2 Florida A & M University; 3 Beth Israel Deaconess Medical Center, Harvard Medical Center; 4 Brigham and Women’s Hospital, Harvard Medical School, Veterans Affairs Boston Healthcare System; 5 Massachusetts Mental Health Center, Beth Israel Deaconess Medical Center, Harvard Medical School; 6 Harvard Medical School, Veterans Affairs Boston Healthcare System; 7 Harvard Medical School

## Abstract

**Background:**

The present study aims to validate the predictive accuracy of the NAPLS-2 psychosis risk calculator in a clinical high-risk (CHR) sample from the SHARP (ShangHai At Risk for Psychosis) program in Shanghai, China using comparable inclusion/exclusion criteria and assessments.

**Methods:**

Three hundred CHR individuals were identified by the Chinese version of the Structured Interview for Prodromal Symptoms. Of these, 228 (76.0%) completed neuro-cognitive assessments at baseline and 199 (66.3%) had at least a one-year follow-up assessment. The latter group was used in risk calculation. Six key predictors (baseline age, unusual thoughts and suspiciousness, symbol coding and verbal learning test performance, functional decline and family history of psychosis) were entered into the NAPLS-2 model to generate a psychosis risk estimate for each case. The area under the receiver operating characteristic curve (AUC) was used to test the effectiveness of this discrimination.

**Results:**

The NAPLS risk calculator showed moderate discrimination of subsequent transition to psychosis in the SHARP sample with an AUC of 0.631 (p = 0.007). Whether discriminating either transition or poor treatment/clinical outcomes, the AUC of the model increased to 0.754 (p < 0.001). A risk estimate of 30% or higher had moderate sensitivity (53%) and excellent speciﬁcity (86%) for prediction of poor treatment/clinical outcome.

**Discussion:**

The NAPLS-2 risk calculator largely generalizes to a Shanghai CHR sample but is meaningfully improved when predicting an individual’s poor clinical outcome as well as conversion. Our findings provide a critical step in the implementation of CHR risk calculation in China.

